# Case report: Endobronchial closure of postoperative bronchopleural fistula with embolization coil: a sandwich-like approach

**DOI:** 10.3389/fmed.2024.1333157

**Published:** 2024-05-13

**Authors:** Yang Bai, Jing Chi, Hansheng Wang, Yishi Li, Shuliang Guo

**Affiliations:** ^1^Department of Respiratory and Critical Care Medicine, The First Affiliated Hospital of Chongqing Medical University, Chongqing, China; ^2^Department of Radiology, The First Affiliated Hospital of Chongqing Medical University, Chongqing, China

**Keywords:** bronchopleural fistula, Embolization Coil, bronchoscopic treatment, case series, interventional pulmonary

## Abstract

**Background:**

Embolization Coil has been reported to effectively treat postoperative bronchopleural fistula (BPF). Little detailed information was available on computer tomography (CT) imaging features of postoperative BPF and treating procedures with pushable Embolization Coil.

**Objective:**

We aimed to specify the imaging characteristics of postoperative BPFs and present our experience treating them with the pushable Embolization Coil.

**Methods:**

Six consecutive patients (four males and two females aged 29–56 years) diagnosed with postoperative BPF receiving bronchoscopic treatment with the pushable Nester® Embolization Coil (Cook Medical, Bloomington, Indiana) were included in this single-center, retrospective study. Multiplanar reconstruction of multidetector CT scans was reviewed for the presence, location, size, and radiological complications of each BPF, including air collection, pneumothorax, bronchiectasis, and chest tube. Using standardized data abstraction forms, demographic traits and clinical outcomes were extracted from the medical files of these patients.

**Results:**

The underlying diseases for lung resection surgery were pulmonary tuberculosis (*n* = 3), lung adenocarcinoma (*n* = 2), and pulmonary aspergillosis (*n* = 1). All patients had air or air-fluid collection with chest tubes on radiological findings. Multiplanar reconstruction identified the presence of postoperative BPF in all patients. Five fistulas were central, located proximal to the main or lobar bronchus, while one was peripheral, distant from the lobar bronchus. Fistula sizes ranged from 0.8 to 5.8 mm. Subsequent bronchoscopy and occlusion testing confirmed fistula openings in the bronchial stump: right main bronchus (*n* = 1), right upper lobe (*n* = 2), and left upper lobe (*n* = 3). The angioplasty catheter-based procedure allows precise fistula occlusion “like a sandwich” with the pushable Embolization Coil. Five patients with BPF sizes ranging from 0.8 to 1.5 mm were successfully treated with a pushable Embolization Coil, except for one with a BPF size of 5.8 mm. No adverse events or complications were observed throughout follow-up, ranging from 29 to 1,307 days.

**Conclusion:**

The pushable Nester® Embolization Coil seems a minimally invasive, cost-effective, and relatively easy-to-perform bronchoscopic treatment for postoperative BPF with a size less than 2 mm. Further studies are required to ensure the use of pushable Embolization Coil in treating postoperative BPF.

## Introduction

Bronchopleural fistula (BPF), categorized as peripheral or central depending on location, is a pathological communication between the main stem, lobar, or segmental bronchus, and the pleural space. It usually results from lung resection surgery, with an incidence of 0.5–1% after lobectomy and 4.5–20% after pneumonectomy, respectively (1). Postoperative BPF can be divided into early (1–7 days), intermediate (8–30 days), or late (> 30 days) onset according to the time of event after the lung resection surgery (2). Postoperative BPF could be a catastrophic complication due to tension pneumothorax or asphyxia from purulent discharge in the thoracic cavity, with an estimated mortality rate of 16.0–71.2% (3). If the clinical condition permits a major reoperation, open thoracotomy or video-assisted thoracoscopic surgery is recommended to repair the postoperative BPF (4). Surgical repair has a documented success rate of over 85% in these individuals with postoperative BPF (5, 6). Bronchoscopic treatments have evolved from a simple diagnostic scheme to a complementary or adjuvant treatment strategy in managing postoperative BPF (7). Bronchoscopic treatments (including sclerosing agent, ethanol injection, sealant, ventricular septal defect occluder, Embolization Coil, etc.) are appropriate for those not candidates for surgical repair, with variable success rates based on the location and size of the fistula (8–13). The pushable Embolization Coil has demonstrated efficacy in treating postoperative BPF with few complications (13–15). However, little detailed information was available on the computer tomography (CT) imaging features of postoperative BPF and treating procedures with pushable Embolization Coil. This report describes the imaging features of postoperative BPFs and presents our experience in treating postoperative BPF with the pushable Embolization Coil.

## Materials and methods

### Patients

Consecutive patients receiving the pushable Embolization Coil for the closure of postoperative BPF were retrospectively recruited in this study from November 1, 2016, to November 1, 2022, at a single academic institution. Most patients failed prior therapies (fibrin sealant and gelatin sponge) and refused the surgical repair. We applied standardized data abstraction forms to collect information on the demographic traits of patients, CT imaging features of postoperative BPF (location, size, and complications), and clinical outcomes of treatment with the pushable Embolization Coil. Patient data were de-identified and complied with the patient confidentiality requirements.

### CT examinations and imaging features

The thin-section or standard contrast-enhanced images were obtained using multidetector CT Scanners, such as the SIEMENS SOMATOM Perspective CT Scanner, SIEMENS SOMATOM Definition DS CT Scanner, GE Discovery CT750 HD CT Scanner, and GE Revolution CT Scanner. The imaging parameters typically employed during CT scanning for BPF evaluation involve using 10–30 mAs for tube current and 100–140 kVp for tube voltage. The mediastinal window was set to a width of 350–450 Hounsfield Units (HU) and a level of 20–40 HU. For the parenchymal window, the width was usually set to 1,200–1,600 HU, while the level ranged from −500 to −700 HU. The postoperative BPF was detected by multiplanar reconstruction from the multidetector with a slice thickness of 1.0 or 0.625 mm on the postprocessing workstation (Picture Archiving and Communication System). Multiplanar reconstruction involves converting axial-plane imaging data into another plane (coronal, sagittal, or oblique). CT scans, especially thin-section CT with multiplanar reconstruction, are cost-effective in directly visualizing and localizing the postoperative BPF (16). The authors (Y.B. and S.H.W.) jointly reviewed the multiplanar reconstruction of CT scans to depict the imaging features of postoperative BPF in this study. Any disagreements during the review were resolved by consensus. CT scans of each patient were assessed for the presence, location (central, proximal to the lobar bronchus, or peripheral, distant), fistula size, and other radiological findings for BPF complications, including air collection, pneumothorax, bronchiectasis, and chest tube. The postoperative BPF on CT scans was defined as a definite fistula tract between the bronchial stump and pleural space (17, 18). We measured the size at the narrowest part of the fistula tract in the reconstructed axial, coronal, sagittal, and oblique planes. Culture-positive fluid or the presence of pus in the pleural space was used to diagnose empyema.

### Techniques

**Figure 1:**
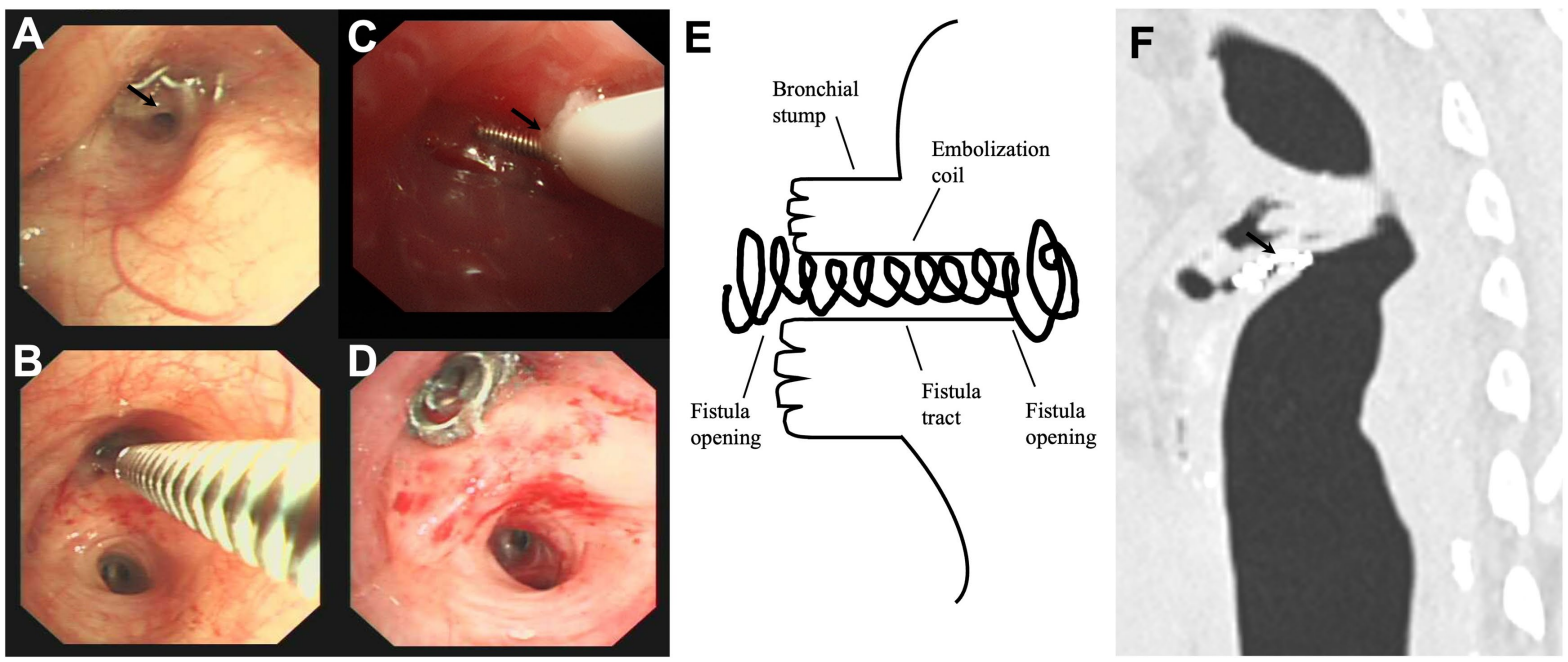
The detailed procedures in treating the postoperative bronchopleural fistula with a pushable Embolization Coil. **(A)** The bronchoscopy found the anastomotic nails and suspected fistula opening (black arrow) in the bronchial stump of the left upper lobe (intrinsic branch). **(B)** Forceps occlusion testing confirmed the fistula opening in the intrinsic branch, stopping the air leak from the chest tube. **(C)** The first half of a pushable Embolization Coil was delivered into the fistula opening via a 5 Fr angioplasty catheter (black arrow). **(D)** The transbronchial insertion of the pushable Embolization Coil successfully occluded the fistula opening, with the other half deployed proximal to the segmental branch. **(E,F)** The schema and chest computed tomography illustrated how the fistula tract was surrounded by the coil’s curves (black arrow) and well-balanced on both fistula openings “like a sandwich”.

The detailed procedures for treating postoperative BPF with a pushable Embolization Coil are illustrated in Figure 1. The flexible bronchoscopy confirmed the location of postoperative BPF and insertion of a dilation balloon (Microtech Co., Ltd., Nanjing, China) or forceps into the segment or subsegment airway with the suspected fistula opening (Figures 1A,B) in patients with persistent air leak (19). If the segment or subsegment airway has a fistula opening, the occlusion testing with the dilation balloon or forceps could obstruct airflow through the fistula tract, reducing or stopping the air leak from the chest tube. The bronchoscope (OLYMPUS, BF-P290) with a 4.1 mm outer diameter and a 2.0 mm working channel was used to assess peripheral postoperative BPF that was more distant than the lobar bronchus. A 5 Fr angioplasty catheter with a pushable Nester® Embolization Coil (Cook Medical, Bloomington, Indiana) was inserted close to the fistula opening (Figure 1C) via the flexible bronchoscope’s working channel. The first half of the Embolization Coil is delivered into the fistula opening using the guidewire, and the rest is deployed proximal to the segmental branch (Figure 1D). The Embolization Coil could be balanced on both fistula openings in the schema and chest CT (Figures 1E,F), mechanically blocking the fistula tract “like a sandwich.” The fibered part could trigger local inflammation, promote granulation tissue formation, and ultimately close the postoperative BPF. Complete closure was defined as the postoperative BPF completely closed with the Embolization Coil without air leak via the chest tube drainage. Incomplete closure was defined as the postoperative BPF partially closed with the Embolization Coil, even using additional coils, fibrin sealant, or both, only with decreased air leak via the chest tube drainage. A chest CT was performed to observe a reduction in residual cavity size and other BPF complications during the follow-up.

## Results

A total of six patients diagnosed with postoperative BPF (four males and two females) aged 29–56 years were consecutively included in this study (Table 1). Two patients underwent pneumonectomy, and four patients underwent lobectomy for pulmonary tuberculosis (*n* = 3), lung adenocarcinoma (*n* = 2), or pulmonary aspergillosis (*n* = 1). Three patients had empyema cultured with different specific organisms, including *Acinetobacter baumannii*, Aspergillus, and Streptococcus pharyngis. All the patients had air collection or air-fluid collection with chest tubes in the radiologic findings at administration. By reconstructing the axial, coronal, sagittal, and oblique planes, the fistula tracts between the bronchial stump and the pleural space were identified in all patients, of which five were central, proximal to the main or lobar bronchus, and one peripheral, distant to the lobar bronchus. The size of the fistula ranged from 0.8 to 5.8 mm. Subsequent bronchoscopy and occlusion testing identified all the fistula openings in the bronchial stump: the right main bronchus (*n* = 1), the right upper lobe (*n* = 2), and the left upper lobe (*n* = 3).

**Table 1:** Characteristics and outcomes in patients with postoperative BPF receiving the treatment of the pushable Embolization Coils.

Patient No./Age (years)/Sex	Clinical history	Organisms	Duration from BPF onset to intervention with coils, months	Radiographic findings	BPF location in CT^*^	BPF tract in CT	BPF size in CT, mm	Bronchoscopic visualization of the fistula opening	BPF location	Microcoils	Outcomes	Follow-up days	Complications
1/46/F	Pulmonary aspergillosis, right pneumonectomy, and persistent air leak	NA	10	Air collection, chest tube	Central	Yes	1.2	Yes	RMB	0.038″ × 3 cm × 4 mm	Complete closure with removal of the chest tube	1,307	No
2/29/M	Pulmonary TB, right upper lobectomy, and persistent air leak	*Acinetobacter baumannii*	24	Air collection, bronchiectasis, and chest tube	Central	Yes	5.8	Yes	RUL	0.035″ × 5 cm × 5 mm × 2	Incomplete closure	988	No
3/51/M	LUAD, left upper lobectomy, persistent air leak	Aspergillus	6	Air collection, Ptx, and chest tube	Central	Yes	1.5	Yes	LUL	0.035″ × 5 cm × 5 mm	Complete closure with removal of the chest tube	782	No
4/50/M	Pulmonary TB, right upper lobectomy, and persistent air leak	NA	6	AF collection, chest tube	Central	Yes	1.0	Yes	RUL	0.035″ × 5 cm × 5 mm	Complete closure	29	No
5/44/F	LUAD *in situ*, left upper lobectomy, and persistent air leak	NA	1	Air collection, Ptx, and chest tube	Central	Yes	0.8	Yes	LUL	0.035″ × 5 cm × 5 mm	Complete closure with removal of the chest tube	82	No
6/56/M	Pulmonary aspergillosis, right pneumonectomy, and persistent air leak	Streptococcus pharyngis	4	Air collection, chest tube	Peripheral	Yes	0.8	Yes	LUL	0.035″ × 5 cm × 5 mm	Complete closure	49	No

AF, Air-fluid; BPF, Bronchopleural fistula; F, Female; LUAD, Lung adenocarcinoma; LUL, Left upper lobe; M, Male; NA, Not applicable; Ptx, Pneumothorax; RMB, Right main bronchus; RUL, Right upper lobe; TB, Tuberculosis; ^*^Central, more proximal than the lobar bronchus or peripheral, more distal than the lobar bronchus.

**Figure 2:**
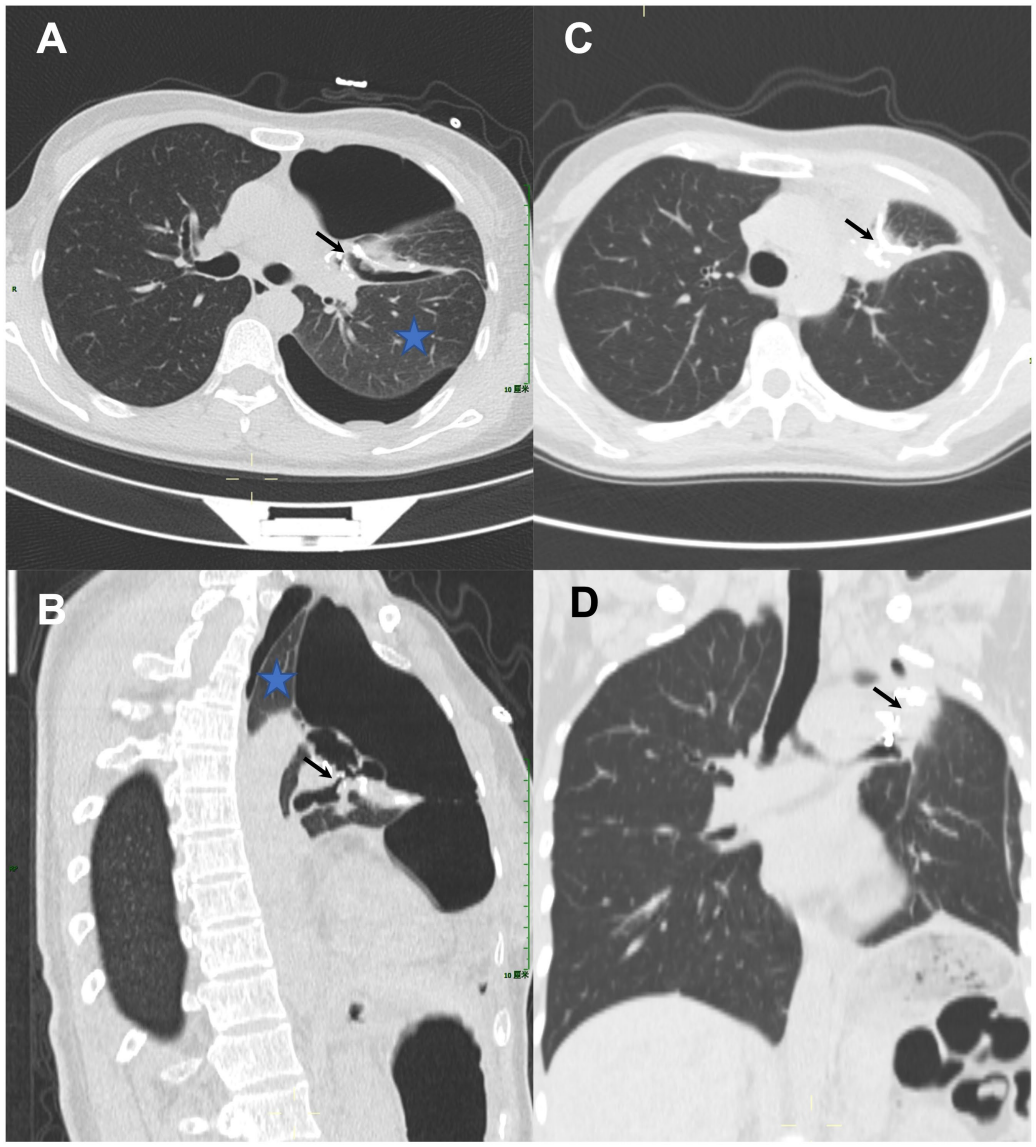
A 44-year-old woman with persistent air leak after left upper lobectomy for lung adenocarcinoma *in situ* received the treatment of a single pushable Embolization Coil. **(A,B)** Chest computed tomography (CT) scans (axial and sagittal planes) demonstrated compressive atelectasis (blue asterisk) and pneumothorax in the left lung, which communicated directly (black arrows) with the bronchial stump. **(C,D)** Follow-up CT scans (axial and coronal planes) obtained 1 month after treatment demonstrated the complete closure of the fistula tract by the curved Embolization Coil which formed a tight occluding mass “like a sandwich” (black arrows), resolving the pneumothorax with the expansion of the collapsed lung tissue.

Figure 2 demonstrates representative CT images of a postoperative BPF before and after a pushable Embolization Coil was inserted. A 44-year-old woman (No. 5) with persistent air leak and chest tube drainage for 1 month after left upper lobectomy for lung adenocarcinoma *in situ* was referred to our department. On chest CT scans, the left lung had both compressive atelectasis and pneumothorax. The pneumothorax was directly connected to the bronchial stump in the axial and sagittal planes (Figures 2A,B). A pushable Embolization Coil (0.035″ × 5 cm × 5 mm) was inserted into the fistula tract as the detailed techniques described in the method part. The chest tube was removed 2 days after the complete closure of the postoperative fistula with the pushable Embolization Coil. Follow-up CT scans obtained 1 month after the treatment demonstrated the complete closure of the fistula tract by the curved Embolization Coil in the axial and coronal planes, resolving the pneumothorax with the expansion of the collapsed lung tissue (Figures 2C,D). Five patients with BPF sizes ranging from 0.8 to 1.5 mm achieved the complete closure of the postoperative BPF with a pushable Embolization Coil alone, three of whom had the chest tube removed and recovered normal life. The other two had persistent chronic empyema, which made permanent drainage necessary. Patient No.2 (with a BPF size of 5.8 mm), who had incomplete closure with the insertion of two pushable Embolization Coils and fibrin sealant, received the placement of a ventricular septal defect occluder. The postoperative BPF was successfully closed, and no complications or adverse events were observed during the follow-up, ranging from 29 to 1,307 days. No patients required the removal of the pushable Embolization Coil. No patients showed any indications of persistent or recurrent underlying diseases during the follow-up.

## Discussion

Postoperative BPF is suspected in patients with persistent air leak through the chest tube and confirmed by chest CT and bronchoscopic evaluation (20). If the peripheral BPF could not be readily identified on CT imaging or by bronchoscopy, additional diagnostic measures should be considered, including bronchography with the water-stable contrast medium, radionuclide studies with Technetium-99 m-labeled albumin or other radiotracers, and surgical exploration (21). In this study, we described the detailed imaging features of postoperative BPF. The multiplanar reconstruction of multidetector CT scans could identify the postoperative BPF’s characteristics, including the BPF’s presence, location, size, and other radiologic findings, which are beneficial in evaluating disease management strategies (17). The BPF size appears to be a significant indicator of the result with bronchoscopic treatment (22, 23). The BPF size over 8 mm (probably with more air leak) was associated with bronchoscopic treatment failure with the adhesive tissue or fibrin glue (21, 24). The dynamic evaluation of BPF size during respiration under bronchoscopy could be challenging due to visualization limitations, dynamic change in BPF size and shape during different phases of respiration, and the lack of standardized measurement tools. The multidetector CT scans used in this study could provide faster image acquisition and improve spatial resolution, reducing the potential for motion artifacts. Additionally, the multiplanar reconstruction in CT imaging allows a more thorough evaluation of the BPF size in different planes (25). It is important to note that bronchoscopy and CT imaging are complementary in evaluating postoperative BPF’s characteristics.

We presented our experience and technical skills with the pushable Embolization Coil in treating postoperative BPF. In this study, the pushable Embolization Coil achieved complete closure of postoperative BPFs with sizes ranging from 0.8 to 1.5 mm, indicating the pushable Embolization Coil might be applied as the initial attempt in treating postoperative BPF with a size less than 2 mm. The pushable Embolization Coil is made of soft platinum reinforced with packed fibers, with wide ranges of diameters and lengths. Embolization Coil could be inserted into the fistula tract through CT-guided transthoracic needles percutaneously, which does not risk pneumothorax but risks bleeding in patients with postoperative BPF (26). We prefer not to insert the pushable Embolization Coil percutaneously in closing postoperative BPF for patients tolerating the bronchoscopy. The angioplasty catheter-based procedure under bronchoscopic observation allows the precise occlusion of the fistula tract with the pushable Embolization Coil, requiring no additional equipment. The fistula tract could be mechanically blocked if the coil is inserted, expanded into it, and balanced on both fistula openings, forming a tight occluding mass “like a sandwich.” The coil could produce a core for the liquid sealants’ occlusion (13, 27, 28). The fibered part could trigger the local inflammation and encourage the growth of granulation tissue formation, completely obstructing the postoperative BPF. This procedure, employing cheap and readily available materials, was minimally invasive without affecting oxygenation in a 2-year-old child (29). The use of volume coils in combination with occlusive materials seems to be a desirable strategy in treating postoperative BPF with large size, allowing a customized approach to match the size and shape of the specific defect.

There are some cases reporting the closure of small BPF with other occlusive materials, such as tissue adhesive, geofoam, fibrin glue, and autologous blood patch (30–33). The forceful pressure generated by a severe cough might dislodge and expectorate these occlusive materials, leading to rapid recurrence of fistula. In some cases, the recurrent fistula after the initial attempts using other occlusive materials was successfully closed with coils (13, 34). Ponn et al. (34) reported the treatment of peripheral BPF with 5-mm Gianturco vascular occlusion coils, in which one coil was dislodged and expectorated 4 months later. None of the patients in this study had coil expectoration because the pushable Embolization Coil could be anchored into the fistula tract as the detailed techniques described in this study. Biological or sclerosing agents can occlude the small BPF by stimulating localized inflammation and scarring, but their effectiveness may vary in various situations (8, 9, 11). None of them have been compared with the pushable Embolization Coil in managing postoperative BPF. Further comparative research is necessary to evaluate the efficacy, complications, and long-term outcomes of different interventions, aiding in determining the optimal management strategy for postoperative BPF.

The study has several limitations that should be addressed. This is a single-center, retrospective study with a small sample size, limiting the generalizability of the findings. The follow-up duration varied among the patients, which did not provide a comprehensive understanding of long-term outcomes. Larger, prospective studies with longer follow-up are needed to draw firm conclusions about the use of pushable Embolization Coil in treating small postoperative BPF.

## Conclusion

We reported a case series of bronchoscopic treatment of postoperative BPF with the pushable Embolization Coil. The pushable Nester® Embolization Coil might be considered the initial bronchoscopic treatment for postoperative BPF with a size less than 2 mm, especially in patients with persistent air leak, pneumothorax, or empyema. It is minimally invasive, cost-effective, and relatively easy to perform with excellent results. More studies are needed to ensure the use of pushable Embolization Coil in treating postoperative BPF.

## Data availability statement

The original contributions presented in the study are included in the article/supplementary material; further inquiries can be directed to the corresponding authors.

## Ethics statement

The studies involving humans were approved by the Institutional Scientific Committee of the First Affiliated Hospital of Chongqing Medical University. The studies were conducted in accordance with the local legislation and institutional requirements. The participants provided their written informed consent to participate in this study. Written informed consent was obtained from the individual(s) for the publication of any potentially identifiable images or data included in this article.

## Author contributions

YB: Conceptualization, Investigation, Writing – original draft, Writing – review & editing. JC: Data curation, Formal Analysis, Writing – review & editing. HW: Data curation, Formal Analysis, Writing – review & editing. YL: Funding acquisition, Project administration, Writing – review & editing. SG: Project administration, Writing – review & editing.

## References

[1] Li S Fan J Liu J Zhou J Ren Y Shen C. Neoadjuvant therapy and risk of bronchopleural fistula after lung cancer surgery: a systematic meta-analysis of 14 912 patients. Jpn J Clin Oncol. (2016) 46:534–46. doi: 10.1093/jjco/hyw037, PMID: 27052116

[2] Le Brigand H. (1973). Fistules bronchiques après pneumonectomies in Appareil Respiratoire, Mediastin, Paroi Thoracique. (ed.) Brigand H Le (Paris: Ed. Masson), XXII: 462–470.

[3] Asamura H Naruke T Tsuchiya R Goya T Kondo H Suemasu K. Bronchopleural fistulas associated with lung cancer operations. Univariate and multivariate analysis of risk factors, management, and outcome. J Thorac Cardiovasc Surg. (1992) 104:1456–64. doi: 10.1016/S0022-5223(19)34643-4 1434730

[4] Bertolaccini L Prisciandaro E Guarize J Spaggiari L. A proposal for a postoperative protocol for the early diagnosis of bronchopleural fistula after lung resection surgery. J Thorac Dis. (2021) 13:6495–8. doi: 10.21037/jtd-21-1095, PMID: 34992827 PMC8662483

[5] Puskas JD Mathisen DJ Grillo HC Wain JC Wright CD Moncure AC. Treatment strategies for bronchopleural fistula. J Thorac Cardiovasc Surg. (1995) 109:995–6. doi: 10.1016/s0022-5223(95)70325-x 7739261

[6] Massera F Robustellini M Pona CD Rossi G Rizzi A Rocco G. Predictors of successful closure of open window thoracostomy for postpneumonectomy empyema. Ann Thorac Surg. (2006) 82:288–92. doi: 10.1016/j.athoracsur.2005.11.046, PMID: 16798231

[7] Sakata KK Reisenauer JS Kern RM Mullon JJ. Persistent air leak—review. Respir Med. (2018) 137:213–8. doi: 10.1016/j.rmed.2018.03.017 29605207

[8] Stratakos G Zuccatosta L Porfyridis I Sediari M Zisis C Mariatou V. Silver nitrate through flexible bronchoscope in the treatment of bronchopleural fistulae. J Thorac Cardiovasc Surg. (2009) 138:603–7. doi: 10.1016/j.jtcvs.2008.10.054 19698843

[9] Takaoka K Inoue S Ohira S. Central bronchopleural fistulas closed by bronchoscopic injection of absolute ethanol. Chest. (2002) 122:374–8. doi: 10.1378/chest.122.1.374, PMID: 12114386

[10] Abramian O Rosenheck J Taddeo-Kolman D Bowen F Boujaoude Z Abouzgheib W. Therapeutic closure of bronchopleural fistulas using ethanol. Ther Adv Respir Dis. (2021) 15:17534666211044411. doi: 10.1177/17534666211044411, PMID: 34494916 PMC8438938

[11] Guo S Bai Y Li Y Chen T. A large central bronchopleural fistula closed by bronchoscopic administration of recombinant bovine basic fibroblast growth factor: a case report. Respiration. (2021) 100:1000–4. doi: 10.1159/000514717, PMID: 34515226

[12] Bai Y Li Y Chi J Guo S. Endobronchial closure of the bronchopleural fistula with the ventricular septal defect occluder: a case series. BMC Pulm Med. (2021) 21:313. doi: 10.1186/s12890-021-01676-3, PMID: 34620149 PMC8496023

[13] Salmon CJ Ponn RB Westcott JL. Endobronchial vascular occlusion coils for control of a large parenchymal bronchopleural fistula. Chest. (1990) 98:233–4. doi: 10.1378/chest.98.1.233, PMID: 2361394

[14] Katoch CD Chandran VM Bhattacharyya D Barthwal MS. Closure of bronchopleural fistula by interventional bronchoscopy using sealants and endobronchial devices. Med J Armed Forces India. (2013) 69:326–9. doi: 10.1016/j.mjafi.2013.04.009, PMID: 24600137 PMC3862617

[15] Marwah V Katoch CDS Kumar K Pathak K Bhattacharjee S Jindamwar P. Bronchoscopic device closure of postoperative bronchopleural fistulae: novel devices and innovative techniques. Lung India Off Organ Ind Chest Soc. (2020) 37:107–13. doi: 10.4103/lungindia.lungindia_179_19, PMID: 32108593 PMC7065536

[16] Gaur P Dunne R Colson YL Gill RR. Bronchopleural fistula and the role of contemporary imaging. J Thorac Cardiovasc Surg. (2014) 148:341–7. doi: 10.1016/j.jtcvs.2013.11.009 24355543

[17] Westcott JL Volpe JP. Peripheral bronchopleural fistula: CT evaluation in 20 patients with pneumonia, empyema, or postoperative air leak. Radiology. (1995) 196:175–81. doi: 10.1148/radiology.196.1.7784563 7784563

[18] Kim EA Lee KS Shim YM Kim J Kim K Kim TS. Radiographic and CT findings in complications following pulmonary resection. Radiographics. (2002) 22:67–86. doi: 10.1148/radiographics.22.1.g02ja0367 11796900

[19] Ratliff JL Hill JD Tucker H Fallat R. Endobronchial control of bronchopleural fistulae. Chest. (1977) 71:98–9. doi: 10.1378/chest.71.1.98 830511

[20] Dugan KC Laxmanan B Murgu S Hogarth DK. Management of Persistent air Leaks. Chest. (2017) 152:417–23. doi: 10.1016/j.chest.2017.02.020, PMID: 28267436 PMC6026238

[21] Lois M Noppen M. Bronchopleural fistulas: an overview of the problem with special focus on endoscopic management. Chest. (2005) 128:3955–65. doi: 10.1378/chest.128.6.3955 16354867

[22] Hollaus PH Lax F Janakiev D Lucciarini P Katz E Kreuzer A. Endoscopic treatment of postoperative bronchopleural fistula: experience with 45 cases. Ann Thorac Surg. (1998) 66:923–7. doi: 10.1016/s0003-4975(98)00589-x, PMID: 9768953

[23] Cardillo G Carbone L Carleo F Galluccio G di Martino M Giunti R. The rationale for treatment of postresectional bronchopleural fistula: analysis of 52 patients. Ann Thorac Surg. (2015) 100:251–7. doi: 10.1016/j.athoracsur.2015.03.014, PMID: 26024752

[24] Shekar K Foot C Fraser J Ziegenfuss M Hopkins P Windsor M. Bronchopleural fistula: an update for intensivists. J Crit Care. (2010) 25:47–55. doi: 10.1016/j.jcrc.2009.05.007, PMID: 19592205

[25] Lee KS Boiselle PM. Update on multidetector computed tomography imaging of the airways. J Thorac Imaging. (2010) 25:112–24. doi: 10.1097/RTI.0b013e3181d7e721, PMID: 20463531

[26] Clemson LA Walser E Gill A Lynch JE Zwischenberger JB. Transthoracic closure of a postpneumonectomy bronchopleural fistula with coils and cyanoacrylate. Ann Thorac Surg. (2006) 82:1924–6. doi: 10.1016/j.athoracsur.2006.01.069, PMID: 17062286

[27] Hirata T Ogawa E Takenaka K Uwokawa R Fujisawa I. Endobronchial closure of postoperative bronchopleural fistula using vascular occluding coils and n-butyl-2-cyanoacrylate. Ann Thorac Surg. (2002) 74:2174–6. doi: 10.1016/s0003-4975(02)04170-x, PMID: 12643416

[28] Sivrikoz CM Kaya T Tulay CM Ak I Bilir A Döner E. Effective approach for the treatment of bronchopleural fistula: application of endovascular metallic ring-shaped coil in combination with fibrin glue. Ann Thorac Surg. (2007) 83:2199–201. doi: 10.1016/j.athoracsur.2007.01.005, PMID: 17532426

[29] Baden W Hofbeck M Warmann SW Schaefer JF Sieverding L. Interventional closure of a bronchopleural fistula in a 2 year old child with detachable coils. BMC Pediatr. (2022) 22:250. doi: 10.1186/s12887-022-03298-y, PMID: 35513808 PMC9074316

[30] Jones DP David I. Gelfoam occlusion of peripheral bronchopleural fistulas. Ann Thorac Surg. (1986) 42:334–5. doi: 10.1016/s0003-4975(10)62748-8, PMID: 3753084

[31] Scappaticci E Ardissone F Ruffini E Baldi S Mancuso M. Postoperative bronchopleural fistula: endoscopic closure in 12 patients. Ann Thorac Surg. (1994) 57:119–22. doi: 10.1016/0003-4975(94)90378-6 8279876

[32] Finch CK Pittman AL. Use of fibrin glue to treat a persistent pneumothorax with bronchopleural fistula. Am J Health Syst Pharm. (2008) 65:322–4. doi: 10.2146/ajhp070101, PMID: 18238769

[33] Wiaterek G Lee H Malhotra R Shepherd W. Bronchoscopic blood patch for treatment of persistent alveolar-pleural fistula. J Bronchol Intervent Pulmonol. (2013) 20:171–4. doi: 10.1097/LBR.0b013e31828f4de0, PMID: 23609256

[34] Ponn RB D'Agostino RS Stern H Westcott JL. Treatment of peripheral bronchopleural fistulas with endobronchial occlusion coils. Ann Thorac Surg. (1993) 56:1343–7. doi: 10.1016/0003-4975(93)90679-c, PMID: 8267434

